# Supplementary material for: A Novel Study of β1- and β2-Adrenergic Receptors Present on PBMCs, T Cells, Monocytes, and NK Cells by Radioligand Method: Quantitation and Correlations
Source: Int J Mol Sci. 2025 Aug 15;26(16):7894. doi: 10.3390/ijms26167894 (PMC12386878; doi:10.3390/ijms26167894)
Supplement: Supplementary file 1 [file ijms-26-07894-s001.zip › File S1.pdf]

Calculation of the content of β_1- and β_2-adrenergic receptors in cells. The content of β1- and β2-adrenoreceptors in cells was calculated on the basis of cell-bound radioactivity as described in [8]. The calculation took into account that the binding of ^{125}I-CYP with cells bearing both types of adrenergic receptors can be decomposed into the following components:

$P = B_1 + B_2 + B_{other} + B_{nonspecific}$

where P is the amount of total radioactivity bound; B_1 is the specific binding to β_1-adrenoreceptors; B_2 is the specific binding to β_2-adrenoreceptors, B_{other} is the specific binding to other cellular receptors (particularly serotonin receptors), and $B_{nonspecific}$ is a completely nonspecific adhesion of ^{125}I-CYP to the cell that is not displaced by high concentrations of unlabeled ligand.

The values of B_{other} and $B_{nonspecific}$ should be the same in the first three cases, as they are unaffected by inhibitor ligands. Assuming that the B_1 value reflects the binding of ^{125}I-CYP to β_1-adrenoreceptors in the absence of inhibitor ligands (measurement I), in case II, this binding would be attenuated to $k_1 \times B_1$, where k_1 is the fraction of residual binding to β_1-adrenoreceptors in the presence of ICI 118,551. For the binding to β_2-adrenoreceptors in case II, we will have $k_2 \times B2$, where k_2 is the fraction of residual binding to β_2-adrenoreceptors in the presence of ICI 118,551. Similarly, in case III, we obtain binding levels $k_3 \times B1$ and $k_4 \times B2$ for β_1 and β_2-adrenoreceptors, respectively (k3 and k4 are the fractions of residual binding to β_1 and β_2-adrenoreceptors in the presence of ICI 118,551 and CGP 20712). The values of k_1-k_4 coefficients were determined experimentally earlier in [8].

If we denote the amount of radioactivity in cpm that bound to cells under the various conditions described in the subsection "Binding Reaction with ^{125}I-CYP" as P1, P2, P3, and P4, then we obtain the following system of equations:

$$P_1 = B_1 + B_2 + B_{other} + B_{nonspecific}$$
$$P_2 = k_1 B_1 + k_2 B_2 + B_{other} + B_{nonspecific}$$
$$P_3 = k_3 B_1 + k_4 B_2 + B_{other} + B_{nonspecific}$$
$$P_4 = B_{nonspecific}$$

From these linear equations, formulas were determined to calculate B_1, B_2 and B_{other} from the data on bound radioactivity, taking into account the k_1-k_4 coefficients.

To calculate the number of adrenergic receptors of each type, approximate formulas corresponding to the chemical equilibrium in the ligand-receptor system were used:

$$R_1 = \alpha \times B_1 \times (1 + K_1/L);\ \ R_2 = \alpha \times B_2 \times (1 + K_2/L),$$

where R_1 and R_2 are the amounts of β_1 and β_2-adrenoreceptors, K_1 and K_2 are the respective binding constants determined previously (K_1 = 40 pM, K2 = 15 pM), L is the concentration of the PC fraction of ^{125}I-CYP: 400000 cpm/ml (at gamma counter efficiency of 79%) or 114 pM under standard assay conditions, α is the conversion factor from cpm to number of molecules (in units), which is 172,000 units/cpm. The content of β_1 and β_2-adrenoreceptors per cell (N_1 and N_2, respectively) is calculated by dividing R_1 and R_2 by the number of cells n in the sample:

$N_1 = R_1/n;\quad N_2 = R_2/n$
