# Supplementary material for: A Novel Study of β1- and β2-Adrenergic Receptors Present on PBMCs, T Cells, Monocytes, and NK Cells by Radioligand Method: Quantitation and Correlations
Source: Int J Mol Sci. 2025 Aug 15;26(16):7894. doi: 10.3390/ijms26167894 (PMC12386878; doi:10.3390/ijms26167894)
Supplement: Supplementary file 1 [file ijms-26-07894-s001.zip › File S2.pdf]

Simultaneous quantitative measurement of ADRB1 and ADRB2 content on the surface of cells (schematic description of the method)

	^{125}I-CYP<br>114 pM<br>80 000 cpm	ICI<br>0.25 µM	CGP<br>0.25 µM	CYP<br>10 µM
1	^{125}I-CYP	---	---	---
2	^{125}I-CYP	ICI	---	---
3	^{125}I-CYP	ICI	CGP	---
4	^{125}I-CYP	---	---	CYP

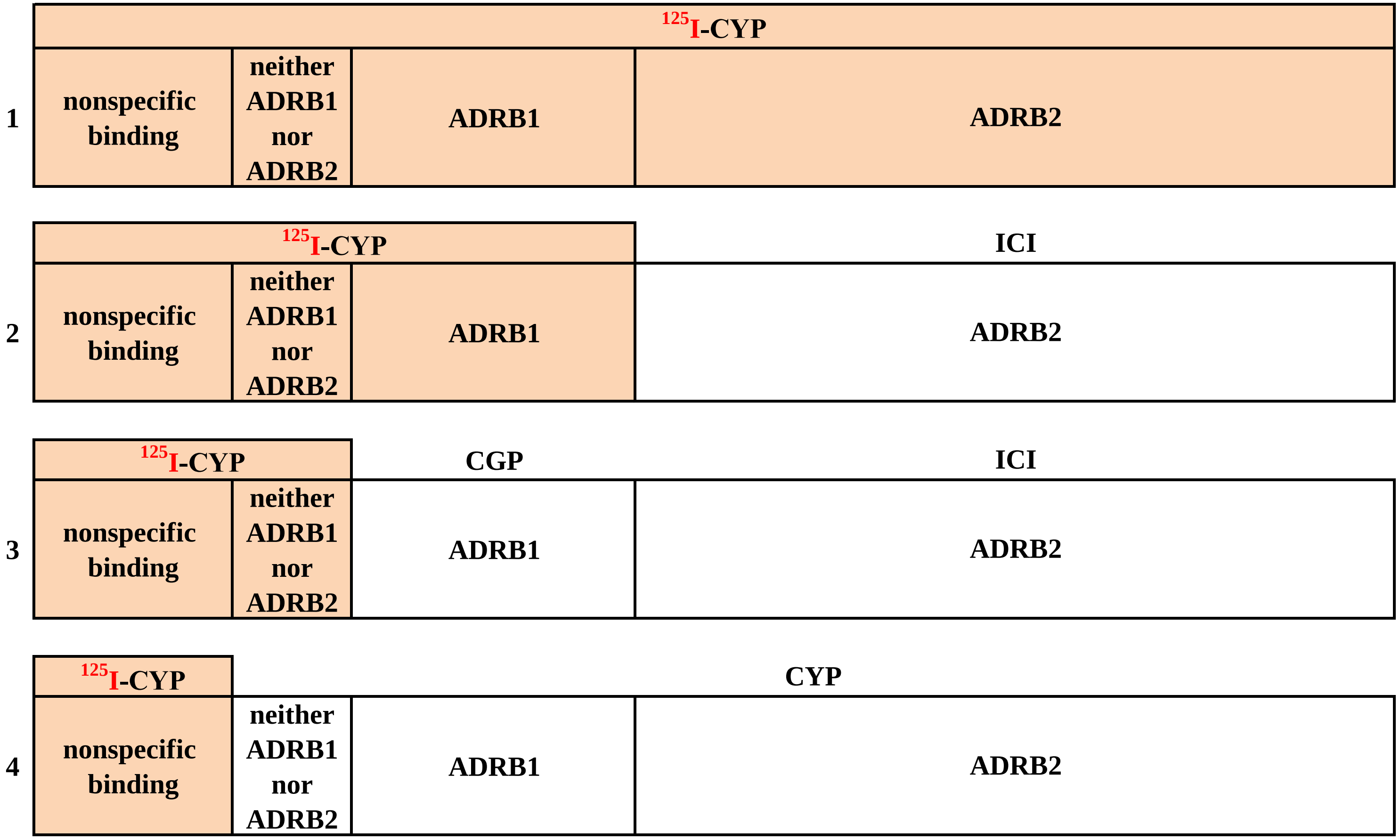

1 minus 2 >>>>> **ADRB2**
2 minus 3 >>>>> **ADRB1**
3 minus 4 >>>>> **neither ADRB1 nor ADRB2**
4 >>>>> **nonspecific binding**
