# Supplementary material for: A Novel Study of β1- and β2-Adrenergic Receptors Present on PBMCs, T Cells, Monocytes, and NK Cells by Radioligand Method: Quantitation and Correlations
Source: Int J Mol Sci. 2025 Aug 15;26(16):7894. doi: 10.3390/ijms26167894 (PMC12386878; doi:10.3390/ijms26167894)
Supplement: Supplementary file 1 [file ijms-26-07894-s001.zip › Table S2.pdf]

Table S4.

The calculated number of ADRB1 and ADRB2 per cell and the standard measurement errors

PBMC			
beta2 Molecules/ cell	SE	beta1 Molecules/ cell	SE
1198	147	0	177
971	36	0	65
302	48	45	91
693	78	0	64
991	312	163	78
1229	106	0	135
1483	113	517	140
1040	76	0	82
1418	161	0	183
569	324	29	261
1058	319	0	78
597	130	0	250
704	109	0	264
567	121	0	296
756	201	0	212
529	141	332	200
692	218	138	242
971	44	0	103
458	51	43	45
221	126	0	265
183	27	0	66
428	19	115	31
52	85	0	119

Monocytes			
beta2 Molecules/ cell	SE	beta1 Molecules/ cell	SE
2091	160	0	148
675	195	300	172
462	53	127	66
556	127	262	161
656	72	130	166
794	209	0	289
880	115	414	149
463	102	0	116
726	108	282	82

453	112	93	61
239	366	1774	127
797	119	0	314
219	279	2215	229
225	203	2174	219
179	269	1672	203
37	143	1122	180
77	120	1406	93
529	65	518	51
225	58	345	46
143	76	0	63
0	85	0	231
251	15	87	56
74	90	0	60

T Cells			
beta2		beta1	
Molecules/ cell	SE	Molecules/cell	SE
974	73	0	23
481	43	0	66
156	66	85	102
389	45	0	26
254	78	65	37
285	66	0	37
1027	175	95	150
341	47	0	38
476	68	0	105
448	55	49	69
175	105	4	106
284	52	0	86
405	85	0	51
119	58	0	88
278	98	0	444
177	131	121	90
93	209	0	47
386	119	0	183
125	17	0	19
40	58	0	40
35	12	0	33
142	11	0	17
82	25	0	73

NK Cells			
beta2		beta1	
Molecules/cell	SE	Molecules/cell	SE

2057	42	148	48
1797	36	0	96
936	53	0	74
711	53	14	57
1981	41	10	43
2943	109	113	283
2560	176	0	111
1579	95	21	72
2375	72	31	109
1434	61	0	53
1103	72	327	112
1311	223	1081	272
1424	77	0	51
861	143	673	114
1184	111	285	73
1411	91	95	65
648	191	655	269
1835	96	300	26
1334	251	0	205
399	67	557	186
620	63	0	172
471	49	20	113
447	85	0	205
